# Fasting plasma triglycerides predict the glycaemic response to treatment of Type 2 diabetes by gastric electrical stimulation. A novel lipotoxicity paradigm

**DOI:** 10.1111/dme.12132

**Published:** 2013-03-07

**Authors:** H E Lebovitz, B Ludvik, I Yaniv, W Haddad, T Schwartz, R Aviv, the Metacure Investigator Group

**Affiliations:** 1 Department of Medicine, State University of New York Health Science Center at BrooklynBrooklyn, NY, USA; 2 Medical University of Vienna, Internal MedicineVienna, Austria; 3 Metacure ResearchRama Gann, Israel; 4 Metacure ResearchVienna, Austria

## Abstract

**Background:**

Non-stimulatory, meal-mediated electrical stimulation of the stomach (TANTALUS-DIAMOND) improves glycaemic control and causes modest weight loss in patients with Type 2 diabetes who are inadequately controlled on oral anti-diabetic medications. The magnitude of the glycaemic response in clinical studies has been variable. A preliminary analysis of data from patients who had completed 6 months of treatment indicated that the glycaemic response to the electrical stimulation was inversely related to the baseline fasting plasma triglyceride level.

**Method:**

An analysis of 40 patients who had had detailed longitudinal studies for 12 months.

**Results:**

Twenty-two patients with fasting plasma triglycerides ≤ 1.7 mmol/l had mean decreases in HbA_1c_ after 3, 6 and 12 months of gastric contraction modulation treatment of −15 ± 2.1 mmol/mol (−1.39 ± 0.20%), −16 ± 2.2 mmol/mol (−1.48 ± 0.20%) and −14 ± 3.0 mmol/mol (−1.31 ± 0.26%), respectively. In contrast, 18 patients with fasting plasma triglyceride > 1.7 mmol/l had mean decreases in HbA_1c_ of −7 ± 1.7 mmol/mol (−0.66 ± 0.16%), −5 ± 1.6 mmol/mol (−0.44 ± 0.18%) and −5 ± 1.7 mmol/mol (−0.42 ± 0.16%), respectively. Pearson's correlation coefficient between fasting plasma triglyceride and decreases in HbA_1c_ at 12 months of treatment was 0.34 (*P* < 0.05). Homeostasis model assessment of insulin resistance was unchanged during 12 months of treatment in patients with high baseline fasting triglycerides, while it progressively improved in patients with low fasting plasma triglycerides. Patients with low fasting plasma triglycerides had a tendency to lose more weight than those with high fasting plasma triglycerides, but this did not achieve statistical significance.

**Conclusions:**

The data presented suggest the existance of a triglyceride lipotoxic mechanism that interferes with gastric/neural mediated pathways that can regulate glycaemic control in patients with type 2 diabetes. The data suggest the existence of a triglyceride lipotoxic pathway that interferes with gastric/neural mediated pathways that can regulate glycaemic control.

## Introduction

Interventional treatments for the management of Type 2 diabetes are gaining wider acceptance. These strategies, which include bariatric surgery, gastric electrical stimulation and endoscopic duodenal sleeve placement, improve glycaemic control and reduce weight in patients with Type 2 diabetes 1–3. The mechanisms responsible for the improvements in glycaemic regulation by these treatments have not been clearly defined and are likely attributable to a variety of different actions, which include weight loss 4, alterations in the secretion of gastrointestinal hormones 5 and changes in central nervous system regulation of food intake and metabolic pathways 6.

Electrical stimulation has been used to cause weight loss in obese individuals with limited and variable success 7. A gastric stimulatory device has been developed that uses gastric contraction modulation to increase glycaemic control and facilitate weight loss in obese patients with Type 2 diabetes 8,9. The stimulatory signal (gastric contractility modulation) has been coupled to device detection of food ingestion. This device, called the TANTALUS II (DIAMOND)®, has been evaluated in several open-label controlled trials and has been shown to improve glycaemic control and cause weight loss in patients with Type 2 diabetes who have inadequate glycaemic control on oral anti-diabetic therapies 8,9. The response to TANTALUS treatment, while always statistically significant, was variable in individual patients. A preliminary analysis of data from the studies unexpectedly showed that the glycaemic response to treatment was related to the fasting plasma triglyceride levels. A detailed analysis of this relationship is the subject of this report.

## Materials and methods

### Patient population

The populations analysed were derived from 40 patients with Type 2 diabetes who had inadequate glycaemic control on oral anti-diabetic agents; had been implanted with the TANTALUS gastric contractility modular device in several almost identical open-label studies; and had follow-up evaluations for a minimum of 12 months. Seventeen of the 40 patients [seven with low triglycerides (fasting plasma triglyceride ≤ 1.7 mmol/l) and 10 with high triglycerides (fasting plasma triglyceride > 1.7 mmol/l] were on statin therapy and one patient in the high triglyceride group was being treated with fenofibrate.

### TANTALUS gastric contractility modulator

**FIGURE 1:**
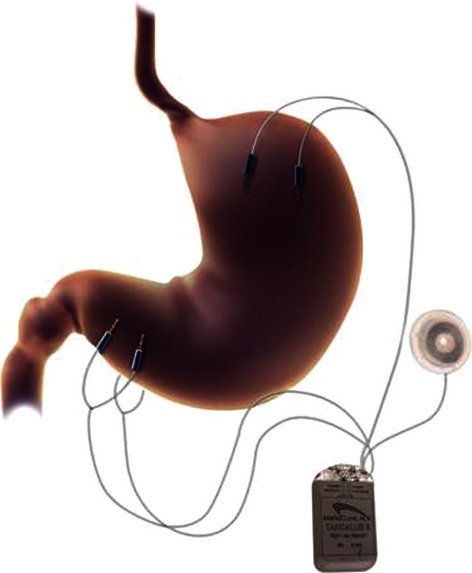
The TANTALUS-DIAMOND device. Three pairs of Tizer electrodes are implanted in the stomach by laparoscopic surgery. The electrodes are externalized and attached to a pulse generator that is placed in a pocket created in the abdominal subcutaneous fat. The fundal electrodes detect nutrient ingestion. They send the signal to the pulse generator, which activates the anterior and posterior antral electrodes. The non-stimulatory impulse increases the force of antral contractions and not the rate. The pulse generator is recharged once a week through the rechargeable port.

The TANTALUS device consists of three pairs of bipolar electrodes and a rechargeable pulse generator, which are implanted, and an external telemetry charger and programmer (Fig. 1). The bipolar electrodes are implanted by laparoscopic surgery: one pair in the fundus used for detection of food ingestion and one pair each on the anterior and posterior antral areas to provide electrical stimulation to the antrum. The electrodes are connected to the pulse generator, which is implanted in a surgically created pocket in the anterior abdominal subcutaneous fat depot. Food ingestion is detected by the fundal electrode that activates the pulse generator to provide the electrical pulse to the antral electrodes. The pulse has unique characteristics (non-excitatory signal) that are set by the programmer during the initial installation of the TANTALUS device in the patient.

### Gastric contractility modulation therapy

Patients with Type 2 diabetes who were on chronic treatment with oral anti-diabetic agents for 6 months or more and had HbA_1c_ > 53 mmol/mol (7%) and < 91 mmol/mol (10.5%) underwent baseline evaluations and were implanted with the TANTALUS device by a laparoscopic surgical procedure. One to 2 weeks after surgery, the pulse generator was programmed to deliver the appropriate signal when food intake was detected by the fundal electrode. Food-mediated stimulation continued for 90 min after activation. The pulse generator battery is recharged weekly for up to 1 h with the external charger. Follow-up data were obtained at 3, 6 and 12 months.

### Measurements

Measurements of glycaemic control (fasting plasma glucose and HbA_1c_) and fasting lipids (triglycerides, total cholesterol, HDL cholesterol and LDL cholesterol) were carried out by the standard laboratory methods. Blood pressures were measured in the sitting position. The homeostasis model assessment of insulin resistance (HOMA-IR) was calculated as described by Matthews *et al*. 10.

### Statistical analyses

Statistical analysis was performed using the SPSS program version 17.0 (SPSS, Chicago, IL, USA). The mean change of HbA_1c_ and the standard error of the mean (sem) were presented as descriptive statistics for all 40 patients who had follow-up evaluation of at least 12 months of treatment. Mean and sem per triglyceride group (low triglycerides ≤ 1.70 mmol/l and high triglycerides > 1.7 mmol/l) of all baseline variables (metabolic and lipids) were calculated for the 40 patients. The change obtained after 6 or 12 months of treatment was calculated from baseline visit. To determine the significance of the change of any variable, the paired student *t*-test was used. Significance level was set at *P* < 0.05.

A histogram plot was presented to show the change of HbA_1c_ and HOMA-IR per each triglyceride group at 6 and 12 months of treatment.

A scatter plot was used to define the relationship between HbA_1c_ and triglyceride at baseline and the decrease after treatment. The Pearson's *R* correlation was used to determine the association between each related variable 11.

## Results

The mean ± sem baseline data for the 40 patients were HbA_1c_ 67 ± 1.5 mmol/mol (8.3 ± 0.12%), body weight 110.5 ± 3.5 kg, systolic blood pressure 139 ± 2.6 mmHg and diastolic blood pressure 86 ± 2.2 mmHg. After 6 months of gastric contraction modulation treatment, the mean HbA_1c_ had decreased by 11 ± 1.4 mmol/mol (−1.0 ± 0.12%, *P* < 0.001), the mean weight by 4.87 ± 0.69% (*P* < 0.01), the systolic blood pressure to 128 ± 3.1 mmHg (*P* < 0.01) and the diastolic blood pressure to 79 ± 2.0 mmHg (*P* < 0.05).

Table 1 presents the baseline data of patients separated into those that had fasting plasma triglycerides within the target range for patients with Type 2 diabetes (≤ 1.7 mmol/l) and those with elevated fasting plasma triglycerides (> 1.7 mmol/l). There was no statistically significant difference in mean HbA_1c_, fasting plasma glucose, body weight, waist circumference, systolic or diastolic blood pressure, total cholesterol, LDL cholesterol or HDL cholesterol between the groups.

**Table 1:** Baseline characteristics of 40 patients with Type 2 diabetes inadequately treated with oral agents: divided into two groups defined by fasting plasma triglycerides ≤ 1.7 mmol/l and > 1.7 mmol/l

	Fasting plasma triglycerides (mmol/l)	*n*	Mean	sem	*P*-value
HbA_1c_ (mmol/mol;%)	≤ 1.7	22	69; 8.4	1.9; 0.24	NS
	> 1.7	18	65; 8.1	1.5; 0.14	
Fasting plasma glucose (mmol/l)	≤ 1.7	20	10.3	0.37	NS
	> 1.7	17	9.9	0.37	
Weight (kg)	≤ 1.7	22	106.8	4.51	NS
	> 1.7	18	115.1	5.49	
Waist (cm)	≤ 1.7	16	121.7	3.5	NS
	> 1.7	13	125.0	3.7	
Systolic blood pressure (mmHg)	≤ 1.7	22	141.7	4.1	NS
	> 1.7	18	135.7	3.0	
Diastolic blood pressure (mmHg)	≤ 1.7	22	85.3	2.9	NS
	> 1.7	18	86.5	3.3	
Total cholesterol (mmol/l)	≤ 1.7	22	4.61	0.23	NS
	> 1.7	18	5.15	0.21	
LDL cholesterol (mmol/l)	≤ 1.7	21	2.91	0.22	NS
	> 1.7	18	2.84	0.20	
HDL cholesterol (mmol/l)	≤ 1.7	21	1.18	0.05	NS
	> 1.7	18	1.21	0.07	

NS, not significant.

**FIGURE 2:**
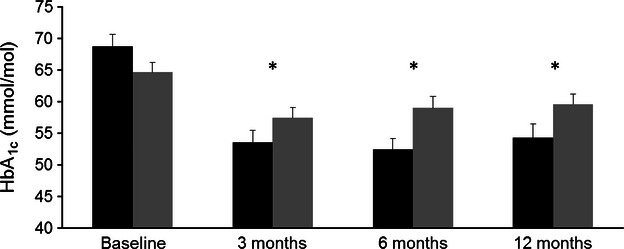
The relationship between the fasting plasma triglyceride (mean baseline and 6 months) and the decrease in HbA_1c_ in patients with Type 2 diabetes inadequately controlled on oral agents. Patients with low triglycerides (black bars, *n* = 22) had fasting plasma triglycerides ≤ 1.7 mmol/l. Patients with high triglycerides (grey bars, *n* = 18) had fasting plasma triglycerides > 1.7 mmol/l. The decreases in HbA_1c_ from baseline in the patients with low triglycerides at 3, 6 and 12 months, respectively, were −15 ± 2.1, −16 ± 2.2 and −14 ± 3.0 mmol/mol (−1.4 ± 0.20,– −1.5 ± 0.20 and −1.3 ± 0.26%) and in the patients with high triglycerides −7 ± 1.7, −5 ± 1.6 and −5 ± 1.7 mmol/mol (−0.7 ± 0.16, −0.4 ± 0.18 and −0.4 ± 0.16%). *P*-values between the groups with low and high triglycerides at 3 and 12 months were 0.008 and at 6 months < 0.0001.

**FIGURE 3:**
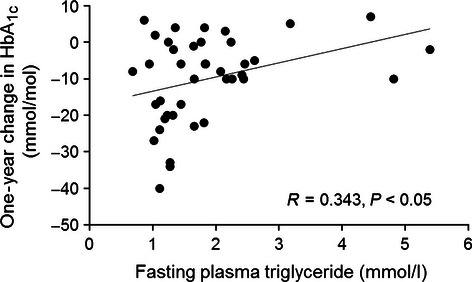
Pearson correlation between fasting plasma triglyceride level and the decrease in HbA_1c_ at 1 year of electrical stimulation treatment in all 40 patients.

The effect of gastric contraction modulation treatment on glycaemic control was strikingly different depending on baseline fasting plasma triglyceride levels. Figure 2 shows the mean HbA_1c_ at baseline and after 3, 6 and 12 months of treatment in those patients with Type 2 diabetes inadequately controlled on oral agents with fasting plasma triglycerides ≤ 1.7 mmol/l (low triglycerides) and > 1.7 mmol/l (high triglycerides). The mean change in HbA_1c_ in those with low triglycerides was −15 ± 2.1 mmol/mol (−1.39 ± 0.20), −16 ± 2.2 mmol/mol (−1.48 ± 0.20) and −14 ± 3.0 mmol/mol (−1.31 ± 0.26%) at 3, 6 and 12 months, respectively. In contrast, the mean change in HbA_1c_ was–−7 ± 1.7 mmol/mol (−0.66 ± 0.16%) at 3 months in the patients with high triglyderides and decreased to −5 ± 1.7 mmol/mol (0.42 ± 0.16%) at 12 months. The relationship between the fasting plasma triglycerides and the HbA_1c_ response to gastric contraction modulation stimulation is shown more clearly in a correlation plot between fasting plasma triglyceride levels and decrease in HbA_1c_ at 12 months (Fig. 3).

The magnitude of HbA_1c_ decrease in response to gastric contraction modulation stimulation is correlated with the baseline HbA_1c_, as has been true with all anti-hyperglycaemic treatments. Table 2 shows that the predictability of the magnitude of HbA_1c_ response to gastric contraction modulation stimulation in the groups with low and high triglycerides s is significant in those patients with baseline HbA_1c_ ≥ 64 mmol/mol (8%) and not discernible in those with HbA_1c_ < 64 mmol/mol (8%). The greatest improvement in glycaemic control with gastric contraction modulation stimulation is seen in patients with low triglycerides and a baseline HbA_1c_ of 64 mmol/mol (8.0%) or greater.

**Table 2:** Decrease in HbA_1c_ (%) as a function of baseline HbA_1c_ and fasting plasma triglycerides

	Baseline HbA_1c_< 64 mmol/mol (< 8%)	Baseline HbA_1c_ 64–75 mmol/mol (8.0–9.0%)	Baseline HbA_1c_ > 75 mmol/mol (> 9.0%)
Low plasma triglycerides	−3.4 ± 3.0 mmol/mol (−0.3 ± 0.2%) (*n* = 8)	−17.9 ± 3.7 mmol/mol (−1.6 ± 0.34%) (*n* = 9)	−26.4 ± 4.6 mmol/mol (−2.4 ± 0.40%) (*n* = 5)
High plasma triglycerides	−3.2 ± 1.9 mmol/mol (−0.3 ± 0.26%) (*n* = 9)	−3.9 ± 2.4 mmol/mol (−0.3 ± 0.26%) (*n* = 7)	−12.5 ± 9.5 mmol/mol (−1.4 ± 0.60%) (*n* = 2)

Gastric contraction modulation stimulation causes a modest weight loss as well as an improvement in glycaemic control. The patients with low triglycerides appear to have a slightly greater weight loss than those with high triglycerides (6 months 5.6 ± 1.1 vs. −4.0 ± 0.8%; 12 months −5.4 ± 1.6 vs. 3.1 ± 0.7%), although the difference was not statistically significant.

While the fasting plasma triglycerides have a significant correlation with the HbA_1c_-lowering effects of gastric contraction modulation stimulation, they do not predict differences in the response of other metabolic variables to gastric contraction modulation stimulation. There was no difference in the responses of waist circumference, fasting plasma glucose, blood pressure or lipids between the groups with low and high triglycerides at 6 and 12 months of treatment. The gastric contraction modulation treatment did not change mean fasting plasma triglyceride levels over the 12 months of treatment. The one variable that did change during therapy and was triglyceride dependent was the HOMA-IR. The HOMA-IR in 11 patients with low triglycerides who had data at baseline and 6 and 12 months was 11.6 ± 1.73, 7.8 ± 1.65 and 5.6 ± 1.16, respectively (*P* < 0.05). In contrast, 11 patients with high trigylcerides with data at baseline and at 6 and 12 months had no significant change from the baseline value of 8.3 ± 1.72.

## Discussion

The TANTALUS device is an effective treatment for improving glycaemic control in patients with Type 2 diabetes not adequately controlled on oral anti-diabetic agents. Previous studies had shown that there was great variability in the magnitude of effect. The present report shows that the level of fasting plasma triglyceride appears to predict the magnitude of the HbA_1c_ improvement. Of the 22 patients with low fasting plasma triglyceride levels, 10 (45.5%) achieved an HbA_1c_ < 53 mmol/mol (7%) at 1 year and seven (31.8%) achieved an HbA_1c_ < 48 mmol/mol (6.5%). In contrast, only three of the 18 patients (16.7%) with high fasting plasma triglycerides achieved an HbA_1c_ < 53 mmol/mol (7%) and one (5.6%) achieved an HbA_1c_ < 48 mmol/mol (6.5%).

The selection of a cut point to distinguish high from low fasting plasma triglyceride levels is arbitrary and to avoid obvious bias we selected the criteria of the upper limit of normal of 1.7 mmol/l, which is generally accepted by the scientific community. The relationship between fasting plasma triglyceride and glycaemic response to the gastric electrical stimulation is in reality a continuous one, as shown by the fasting plasma triglyceride vs. decrease in HbA_1c_ plot depicted in Fig. 3. A potential confounding issue with our data is the stability of the fasting plasma triglyceride levels during the year of study. One might anticipate that the fasting plasma triglyceride in the patients might change significantly during the course of treatment for 1 year and that this would have an effect on the results of the gastric contraction modulation treatment during the year. The fasting plasma triglyceride values used for our analyses were the mean of the values at baseline and after 6 months of treatment. For the group with low triglycerides, both values were < 1.7 mmol/l in 20 of the 22 patients. The means ± sem for the fasting plasma triglycerides for the entire group with low triglycerides were: baseline 1.25 ± 0.05, 6 months 1.16 ± 0.07 and 12 months 1.26 ± 0.15 mmol/l. For the group with high triglycerides, the two values were > 1.7 mmol/l in 14 of 18 patients and the means ± sem for the entire group were at baseline 2.65 ± 0.26, at 6 months 2.52 ± 0.40 and at 12 months 2.20 ± 0.32 mmol/l.

The present report describes a unique association between the glycaemic response to a therapy for Type 2 diabetes and fasting plasma triglyceride levels. Gastric contractility modulation stimulated by food ingestion decreases HbA_1c_ and body weight in patients with Type 2 diabetes who are inadequately controlled on combinations of oral agents. Gastric contraction modulation reduces fasting hyperglycaemia and postprandial glucose excursions and causes modest weight loss. Studies indicate that it decreases blood pressure, increases early meal-mediated insulin secretion and suppresses glucagon secretion. These effects suggest a central nervous system-mediated action that is similar to but not identical to that of glucagon-like peptide 1 (GLP-1) receptor agonists.

The triglyceride effect shown in the present data could be explained by a direct effect of triglycerides to inhibit the glycaemic effects of the gastric contraction modulation. This has not been noted with other anti-hyperglycaemic agents or procedures. Another possibility could be that high triglycerides are markers for some other metabolic abnormality which occurs in these patients with high triglycerides and that abnormality might block the mechanism responsible for the glycaemic-lowering effect of gastric contraction modulation. For example, pioglitazone has been shown to decrease fasting glucose and mean peak postprandial glucose levels in a manner that is closely correlated with its effects in decreasing hepatic triglycerides 12. In obese individuals, liver, muscle and adipose tissue insulin action has been shown to correlate with intrahepatic triglyceride content 13. Whether the effect of high triglyceride levels in decreasing the glycaemic response to gastric contraction modulation is a direct effect can be determined by a properly designed intervention study with a triglyceride-lowering agent vs. a placebo in patients with Type 2 diabetes with inadequately controlled glycaemia treated with gastric contraction modulation.

There are considerable data concerning the role of lipotoxicity in the pathogenesis and progression of Type 2 diabetes 14,15. Increases in intracellular lipids (free fatty acids, triacylglycerol- and triglyceride-rich lipoproteins) generate metabolic products such as diacylglycerols, long-chain acyl-CoAs and ceramides 16,17. An excess in intracellular lipids in skeletal muscle, liver and heart muscle results in insulin resistance and altered organ function 17–19. There is strong evidence that lipotoxicity is an important factor in increasing β-cell apoptosis and decreasing β-cell insulin secretion in cell cultures and rodent models 20,21. The effects of increased lipids in human β-cell function are unclear. Infusions of triglycerides with heparin raise plasma free fatty acids and have been used to study lipotoxicity in humans. Raising plasma free fatty acids for several days in control humans has been reported to have no effect or to cause an increase in β-cell insulin secretory function 22,23. Similar studies in individuals with pre-diabetes leads to decreased insulin secretory function 24. In patients with Type 2 diabetes, comparable studies show no effect or a modest decrease in β-cell insulin secretory function 23.

Studies in experimental animals have identified both an intestine–brain–liver axis and a brain–liver axis by which long-chain fatty acid acyl-CoA levels suppress hepatic glucose production 25–27. In a series of elegant experiments, Wang *et al*. 25 demonstrated that increases in long-chain fatty acid acyl-CoA in the upper intestine stimulate neural signal transmission via the afferent vagus nerve to the nucleus of the solitary tract in the hindbrain, where a signal is subsequently generated that traverses the hepatic branches of the efferent vagus nerve and increases insulin sensitivity and decreases hepatic glucose production. This intestine–brain–liver pathway presumably reduces glucose production in response to the lipid content of the meal. A distinct brain–liver axis involves the sensing of circulating long-chain fatty acid acyl-CoA by the arcuate nucleus of the hypothalamus 26. This activates hypothalamic adenosine-5'-triphosphate (ATP)-dependent potassium channels. The result is the generation of a chain of neuronal events that cause signalling through selective centres in the brain stem to the descending fibres of the hepatic branch of the vagus nerve to the liver, leading to suppression of hepatic glucose production 27. Factors which interfere with these pathways could lead to an inability to suppress hepatic glucose production during exposure to ingested lipids. Additionally, abnormalities of the hypothalamic ATP-dependent channel activity decrease the ability of central or peripheral insulin to decrease hepatic glucose production 27. Our data could be explained by an effect of chronic hypertriglyceridemia in attenuating a presumed effect of gastric contraction modulation stimulation on the hypothalamic regulation of hepatic glucose production during feeding.

There do not appear to be any reports of a modulating effect of either plasma triglycerides or plasma free fatty acid levels on the glycaemic response to other therapeutic agents or devices. The Miami transplant unit, however, recently reported a relationship between baseline fasting plasma triglyceride levels and pancreatic islet graft survival in 44 recipients of islet transplantation 28. Higher baseline triglycerides were associated with earlier decline in islet graft function. No such relationship was noted with total, LDL or HDL cholesterol levels.

Our observations of an inverse relationship between the improvements in glycaemia with the fasting plasma triglyceride levels in gastric contractility modulatory therapy raises many questions. Is there a relationship between fasting plasma triglycerides and other anti-hyperglycaemic agents? Are the effects that we have observed a manifestation of a specific lipotoxic mechanism or are they attributable to some other metabolic factor that is associated with fasting plasma triglycerides. The answers to these questions could provide new insights into the complex interactions associated with the therapy of Type 2 diabetes.

A practical outcome of the observations on fasting plasma triglycerides and baseline HbA_1c_ presented is the potential ability to predict which patients with Type 2 diabetes who are inadequately controlled with oral agents are likely to have a significant benefit from treatment with gastric contraction modulation stimulation. If the effects of high triglycerides are directly related to the triglycerides or their metabolic products, it may be possible to increase the benefits of gastric contraction modulation stimulation by pretreating such patients with triglyceride-lowering agents.

### Funding sources

This article is funded by Metacure Inc., Rama Gann, Israel.

### Competing interests

HEL has received travel reimbursements and honoraria from Metacure Inc. for attending several conferences and chairing its Senior Medical Advisory Committee. HEL holds stock options for Metacure Inc. BL has received travel reimbursement and honoraria from Metacure Inc. for attending and speaking at conferences, funds for research and honoraria for consulting. IY is an employee of Metacure Inc. and has shares in the company. WH is a former employee of Metacure Inc. and has shares in the company. TS is an independent statistical consultant and holds shares in Metacure Inc.
